# Validation of AshTest as a Non-Invasive Alternative to Transjugular Liver Biopsy in Patients with Suspected Severe Acute Alcoholic Hepatitis

**DOI:** 10.1371/journal.pone.0134302

**Published:** 2015-08-07

**Authors:** Marika Rudler, Sarah Mouri, Frederic Charlotte, Philippe Cluzel, Yen Ngo, Mona Munteanu, Pascal Lebray, Vlad Ratziu, Dominique Thabut, Thierry Poynard

**Affiliations:** 1 Assistance Publique Hôpitaux de Paris, University Pierre et Marie Curie, Paris, France; 2 BioPredictive, Paris France; 3 INSERM UMRS 938, Paris, France; Institute of Medical Research A Lanari-IDIM, University of Buenos Aires-National Council of Scientific and Technological Research (CONICET), ARGENTINA

## Abstract

**Background/Aims:**

According to guidelines, the histological diagnosis of severe alcoholic steatohepatitis (ASH) can require liver biopsy if a specific treatment is needed. The blood test AshTest (BioPredictive, Paris, France) has been initially validated for the non-invasive diagnosis of ASH in a large population of heavy drinkers. The aim was to validate the AshTest accuracy in the specific context of use of patients with suspected severe ASH, in order to reduce the need for transjugular biopsy before deciding treatment.

**Methods:**

The reference was liver biopsy, performed using the transjugular route, classified according to its histological severity as none, minimal, moderate or severe. Biopsies were assessed by the same experienced pathologist, blinded to simultaneous AshTest results.

**Results:**

A total of 123 patients with severe clinical ASH (recent jaundice and Maddrey function greater or equal to 32) were included, all had cirrhosis and 80% had EASL histological definition of ASH. 95% of patients received prednisolone; and the 2-year mortality was 63%. The high AshTest performance was confirmed both for the binary outcome [AUROC = 0.803 (95%CI 0.684–0.881)] significantly higher than the AST/ALT AUROC [0.603 (0.462–0.714); P<0.001], and for the severity of ASH-score system by the Obuchowski measures for [mean (SE) 0.902 (0.017) vs. AST/ALT 0.833 (0.023); P = 0.01], as well as for the diagnosis and severity of ballooning, PMN and Mallory bodies. According to attributability of discordances, AshTest had a 2–7% risk of 2 grades misclassification.

**Conclusion:**

These results confirmed the diagnostic performance of AshTest in cirrhotic patients with severe clinical ASH, in the specific context of use of corticosteroid treatment. AshTest is an appropriate non-invasive alternative to transjugular liver biopsy.

## Introduction

In patients with suspected alcoholic liver disease, recent guidelines recognized that the precise indications of liver biopsy are not well established in routine practice due to significant morbidity/mortality related to liver biopsy. There is however a consensus that transjugular liver biopsy "should be considered" for the diagnosis of severe alcoholic steatohepatitis (ASH) that is amenable to specific therapy such as corticosteroids, both in the EASL and AASLD guidelines,[1,2] These guidelines have also recommended studies validating diagnostic algorithms including liver biopsy and non-invasive tests.[1,2]

In 2006 we published the first accuracy validation study of a non-invasive biomarker called AshTest (BioPredictive, Paris, France).[3] Two hundred and twenty-five heavy-drinker patients were included: 70 in the training group, 155 in the validation groups, and 299 controls. AshTest was constructed using a combination of the six components of FibroTest–ActiTest plus aspartate aminotransferase (AST). The AshTest area under the ROC curves (AUROC) for histologically moderate to severe ASH was 0.90 in the training group, and 0.88 to 0.89 in the validation groups. One limitation of this study was the relatively small number of patients with severe histological ASH (n = 53).

The specific aim of the present study, in comparison to the first validation, was to focus on the performance of AshTest in the real context of use, which is a patient with suspected severe clinical and histological ASH who requires specific treatment such as corticosteroid. The following performances of AshTest as a surrogate have been assessed: be in the diagnosis of mild, moderate and severe histological steatohepatitis, in the binary diagnosis of histological ASH according to different definitions, and the correlation with ASH score.

## Patients and Methods

AshTest was prospectively assessed in consecutive patients admitted to the Hepatology department’s intensive care unit at "Groupe Hospitalier Pitié Salpêtrière" for suspected severe ASH, clinically defined in a patient with severe liver dysfunction in the context of excessive alcohol consumption and the exclusion of other causes of acute and chronic liver disease, [1,2] such as advanced hepatocellular carcinoma and other etiologies of cirrhosis. Severe liver disease was defined as "Severe liver disease was defined as jaundice in the past 3 months, Maddrey discriminant function (DF) ≥32 at admission and a total serum bilirubin level >50 mol/L. During hospitalization, patients with clinical complications, such as ascites, spontaneous bacterial peritonitis, renal dysfunction, overt hepatic encephalopathy, or gastrointestinal bleeding associated with portal hypertension, were treated according to current international guidelines.[1,2]

This non-interventional study was exempt from IRB review after institutional IRB review (Ethical committee of "Comité de Protection des Personnes of Paris- Ile-de-France", FIBROFRANCE-project. CPP-IDF-VI, 10-1996-DR-964, DR-2012-222, and USA-NCT01927133). All data were analyzed anonymously. All clinical investigation were conducted according to the principles expressed in the Declaration of Helsinki.

We ask to all patients of our unit to sign an inform consent for this type of utilization of blood sample. All co-authors had access to the study data and had reviewed and approved the final manuscript.

### Histology

Liver biopsies were fixed, paraffin-embedded and stained with hematoxylin–eosin–safran and Masson’s trichrome or picrosirius red for collagen. A single, experienced pathologist (FC), unaware of the patient characteristics, including biomarker results, analyzed the histological features using a specific scoring system, at x200 and x400 magnification.

Scoring procedures that focus on the main "independent" elementary lesions, as proposed for chronic viral hepatitis [4] or non-alcoholic steatohepatitis (NASH) [5,6], could be readily adapted for use in histological ASH.[7] We previously used such scoring systems in these patients with ASH by accumulating the grades of the elementary ASH lesions.[3,8] For consistency with these recommendations, we used two primary endpoints: one binary (the presence or absence of ASH) and one non-binary (ordinal according to a score) endpoint.

Histological ASH was defined as the presence of steatosis, ballooning and PMN (EASL definition),[2] In sensitivity analyses, the following four other definitions of ASH were used: the pathologist’s main conclusion, the most sensitive definition presence of at least one elementary activity feature (ballooning, PMN or Mallory bodies), and the most specific that is presence of all three features.

The non-binary primary endpoint (ordinal), was calculated in the identical fashion as the NAS score. The sum of the three elementary lesion grades (none, minimal, moderate, severe; from 0 to 3), resulting in a 4-grade severity score: H0, no ASH; H1, minimal ASH (score 1–2); H2, moderate ASH (score 3–5); and H3, severe ASH (score 6–9). In sensitivity analyses, the severity of histological ASH, also given by the pathologist in four grades in his conclusion, was also used to evaluate the test performance.

An international multicenter study proposed, as a prognosis index, a new histological classification of ASH (the AHHS [Alcoholic Hepatitis Histologic score], from 0 to 9: mild 0–3; intermediate 4–5; severe 6–9).(**S1 File**).[9] The prognostic performances of this score were also "retrospectively" compared to AshTest.

Steatosis was scored from 0 to 100 according to the percentage of hepatocytes with macro- or microsteatosis. Fibrosis was staged with a scoring system adapted from the METAVIR score using a scale from F0 to F4.[10]

### Serum biochemical biomarkers

AshTest was performed according to the analytical recommendations and analyzed using the same cutoffs as in the previous studies. AshTest (BioPredictive, Paris, France), combined the six components of the FibroTest–ActiTest, [GGT, ALT, total bilirubin (BILI), alpha2-macroglobulin (A2M), apolipoprotein A1 (APOA1), and haptoglobin (HAPTO)] with the serum AST activity and specific algorithms adjusted for age and gender. AshTest scores range from 0 to 1.00, with higher scores indicating a greater probability of significant lesions. FibroTest and SteatoTest (BioPredictive, Paris, France; FibroSURE LabCorp, Burlington, NC, USA) were determined using published recommended pre-analytical and analytical procedures.[3,11]

GGT, ALT, AST and BILI were measured by a Hitachi 917 analyzer and Roche Diagnostics reagents (both Mannheim, Germany), A2M, APOA1, and HAPTO were measured using a Modular analyzer (BNII, Dade Behring; Marburg, Germany). All coefficient of variation assays were lower than 10%.

### Analyses of discordances

Discordance between biopsy and biomarker tests for the prediction of histological ASH was analyzed according to their respective risk factors of failure.[3,11] Risk factors of AshTest failure were hemolysis, Gilbert’s disease, acute inflammation and extra hepatic cholestasis, together with extreme values of the respective components (haptoglobin, bilirubin, GGT). Patented AshTest as FibroTest, includes algorithms which automatically exclude profile of components at high-risk of false positive/negative, the test being classified as not reliable.[11]

According the obvious risk of commercial bias we performed several analyses of association between risk of false positive/negative and specific conditions of severe ASH, which could interact with AshTest components. We compare the value of AshTest and its components between patients with or without sepsis at admission to identify if infected patients had an inflammatory profile with increasing haptoglobin (risk of false negative) or A2M (risk of false positive) in comparison without sepsis.

High risk factors of biopsy failure were specimen length and fragmentation: less than or equal to 15 mm long, or between 15 and 19 mm but with more than 10 fragments. Failure was attributable to biopsy when there was no high risk of AshTest failure and if the biopsy specimen was at high risk of failure.

Due to the risk of commercial bias another analysis was made as the worse scenario for AshTest and AST/ALT, assuming that biopsy was a perfect reference without any false positive/negative.

### Hemodynamic study

After fasting overnight, patients were placed in the supine position, and the wedged and free hepatic venous pressure gradients [HVPG] were measured by two experienced operators using an 8F catheter (Cordis SA, Miami, FL, USA) inserted into the right hepatic vein. Two similar consecutive values of the HVPG were used. PHT (portal hypertension) was defined as an HVPG ≥5 mmHg, and severe PHT as an HVPG ≥12 mmHg.[12]

### Statistical analyses

The two primary outcomes (binary and non-binary) were predetermined.

The binary outcome was the presence of ASH, the standard definition being steatosis, ballooning and PMMN.[2] The AUROCs were estimated by the empirical (nonparametric) method of Delong *et al*. and compared using the paired method [13];

The ordinal (non-binary) AshTest cutoffs were predetermined (US patent 7856319 B2) ≤0.1700 (no ASH), ≤0.5535 (minimal), ≤0.780 (moderate), and >0.780 (severe ASH). The non-binary diagnostic performances of AshTest (or standard tests) used the Obuchowski measures to prevent the risk of spectrum effect and to reduce the risk of multiple testing.[14] The Obuchowski measure allows two biomarkers to be compared with a single test, avoiding appropriate correction for the type I error when comparing two biomarkers for different stages or grades.(**S2 File)** Obuchowski measure is a multinomial version of the AUROC. The overall Obuchowski measure is not equivalent to a usual AUROC curve, as the measurements are weighted according to the distance between grades.

Sensitivities, specificities, and positive (PPV) and negative predictive values (NPV) were assessed according to predetermined cutoffs.

Sensitivity analyses compared AUROCs according to biopsy specimen length and the number of fragments, and according to several definitions of histological ASH based on combinations of elementary lesions. False positive and false negative results of AshTest were defined using a previous cutoff of 0.50 for the binary endpoint. and analyzed with AUROCs.

There is no reference test, and the diagnostic performance of AshTest was compared with the standard AST/ALT ratio, a non-patented quantitative score, and for prognostic performance with DF function, the MELD score, and FibroTest.

For the discordance analyses, the weighted quadratic kappa (wKappa) and the maximum-adjusted Kappa with observed marginal totals were assessed to identify possible variability factors, such as biopsy specimen length or fragmentation.

The following methods were used when appropriate: the chi-squared, Fisher’s exact test for qualitative comparisons, Student’s t-test, Z-test and the Mann–Whitney U test for quantitative comparisons. For survival analyses, time-dependent Kaplan–Meier analysis for survival curves, the log-rank test for univariate comparisons and the Cox proportional hazard model for multivariate analysis were performed. For all analyses, two-sided statistical tests were used; a P-value of 0.05 or less was considered significant. Statistical analyses were performed using Number Cruncher Statistical Systems 2012 software (NCSS, Kaysville, Utah, USA) and R software [library(nonbinROC) and library(ROCR)].[15,16].

## Results

**Fig 1:**
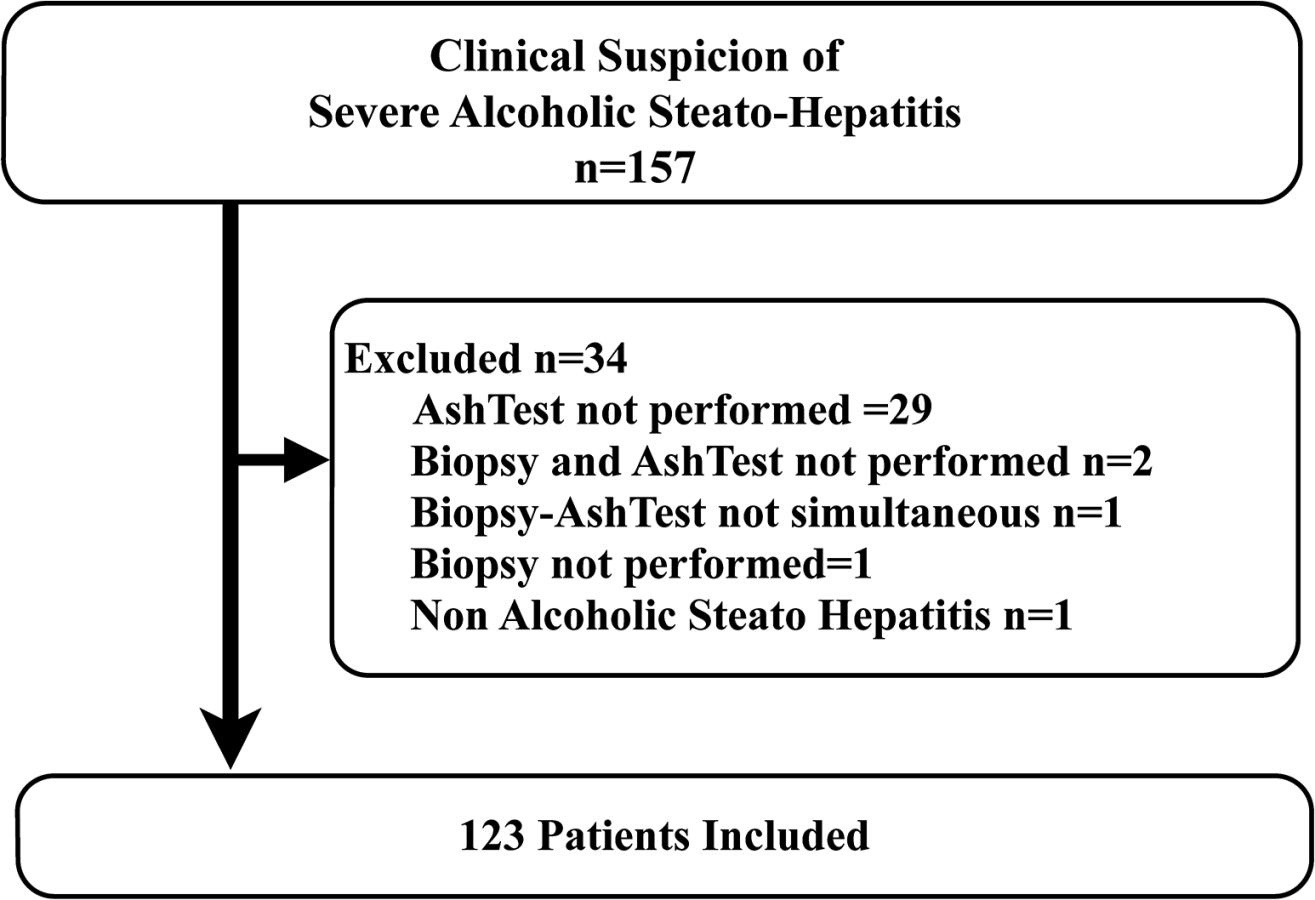
Flow chart of inclusion.

Between 2004 and 2013, of 157 eligible patients, 123 were included and 34 not included. In 31 patients AshTest was not performed due to forgotten prescription or too small blood sample. Among the 123 included patients, AshTesT and biopsy were obtained the same day except for one case obtained 3 days apart. (Fig 1).

There were no significant differences in the main patient characteristics.(**Table 1**) According to the definition, the prevalence of histological ASH varied from 78% (ballooning and PMN, and Mallory), 80% for the EASL definition (Ballooning and PMN and steatosis), to 91% for at least one activity lesion (Ballooning or PMN or Mallory).**(S1 Table)** All patients had cirrhosis both observed at histology and presumed by FibroTest (median 0.98; range 0.80–1.00); one FibroTest was not interpretable which can be considered as a failure in intention to diagnose.

**Table 1:** Comparison between included and non-included patients.

Baseline characteristics	Included (n = 123)	n	Non-included (n = 34)	n	P-value
**Male**	92 (75.0%)	123	25 (74%)	34	1.00
**Age mean (sd)**	56.7 (8.8)	123	52.4 (10.5)	34	0.03
**Hemorrhage**	61 (50.0%)	123	17 (50%)	34	1.00
**Baseline sepsis**	23 (18.7%)	123	9 (26.5%)	34	0.31
**Small hepatocellular carcinoma**	5 (4.1%)	123	3 (8.8%)	34	0.37
**Child C**	109 (88.6%)	123	31 (91.2%)	34	1.00
**Corticosteroids**	117 (95.1%)	123	31 (91.2%)	34	0.41
**Death**	78 (63.4%)	123	19 (55.9%)	34	0.43
**Transplantation**	11 (8.9%)	123	5 (14.7%)	34	0.34
**Indexes**					
**Maddrey function**	62.7 (31.4)	123	58.7 (23.5)	28	0.56
**MELD score**	20.7 (7.0)	123	20.7 (7.7)	27	0.91
**AST/ALT**	2.8 (1.5)	123	3.1 (1.6)	3	0.62
**FibroTest**	0.96 (0.04)	122^1^	0.73 (0.32)	4	0.005
**AshTest**	0.76 (0.27)	123	0.44 (0.38)	4	0.05
**Biopsy**					
**Duration test-biopsy (day)**	0 (0.05))	123	0 (0.01)	28	0.58
**Wedged hepatic venous pressure gradient (mm Hg)**	16.9 (8.6)	97	17.3 (8.8)	9	0.85
**Inferior vena cava (mm Hg)**	13.7 (6.5)	97	9.4 (5.0)	9	0.03
**Length specimen (mm)**	15.4 (6.1)	123	14.3 (4.6)	25	0.62
**Less or equal to 15mm**	53 (43.1%)	123	9 (46.4%)	25	0.83
**Number of fragments**	10.3 (5.8)	123	10 (6.1)	28	0.96
**More or equal to 10**	53 (43.1%)	123	13 (46.4%)	28	0.83
**Risk of biopsy failure**	84 (68.3%)	123	20 (80%)	25	0.18
**Cirrhosis**	123 (100%)	123	28 (100%)	28	1.00
**Histological ASH definition**					
Ballooning and PMN and Mallory	96 (78.0%)	123	21 (75%)	28	0.80
Ballooning and PMN and steatosis (EASL definition)	98 (79.7%)	123	12 (42.9%)	28	0.01
Mallory bodies	103 (83.7%)	123	21 (75%)	28	0.27
Ballooning and PMN	101 (82.1%)	123	23 (82.1%)	28	1.00
**Ballooning**	106 (86.2%)	123	24 (85.7%)	28	0.77
**PMN**	106 (86.2%)	123	23 (82.1%)	28	0.55
**Pathologist binary conclusion**	106 (86.2%)	123	23 (82.1%)	28	0.56
**Ballooning or PMN or Mallory**	112 (91.1%)	123	24 (85.7%)	28	0.48

^1^One FibroTest was not reliable, and all AshTest were reliable

No AshTest with high-risk of false positive/negative was identified by algorithms. No case of elevated unconjugated bilirubin or extra-hepatic cholestasis was identified at the ultrasonography performed in all patients and no severe hemolysis was identified. The proteins included in AshTest and possibly associated with acute or chronic inflammation were not associated with sepsis at baseline, such as haptoglobin (mean (SD)), and alpha2-macroglobulin: 0.89 g/L (0.12) and 1.84 g/L (0.74) among the 23 patients with sepsis vs 0.73 (0.28) and 1.99 (0.70) in 100 patients without sepsis at baseline (P = 0.28 and P = 0.29) respectively. Only the MELD prognostic index was associated with sepsis, as well as a dramatic decrease in apoA1 as previously observed.[3]**(S2 Table)**

Biopsy specimens had a risk of failure in 84/123 (68%) patients, with medians of 10 mm in length and 10 fragments. A severe adverse event, possibly associated with transjugular biopsy, occurred in one patient (0.8%). Acute respiratory distress syndrome (desaturation and bilateral lung opacity) occurred one hour after the biopsy together with a generalized seizure. The patient was intubated and treated successfully in the ICU with nitric oxide, intravenous corticosteroid and antibiotics. No hematoma, pneumothorax or capsular perforation was observed; transthoracic ultrasonography was normal, and no infection was identified. Ventricular arrhythmia was not ruled out as a possible cause.

### Diagnostic performance of AshTest

**Fig 2:**
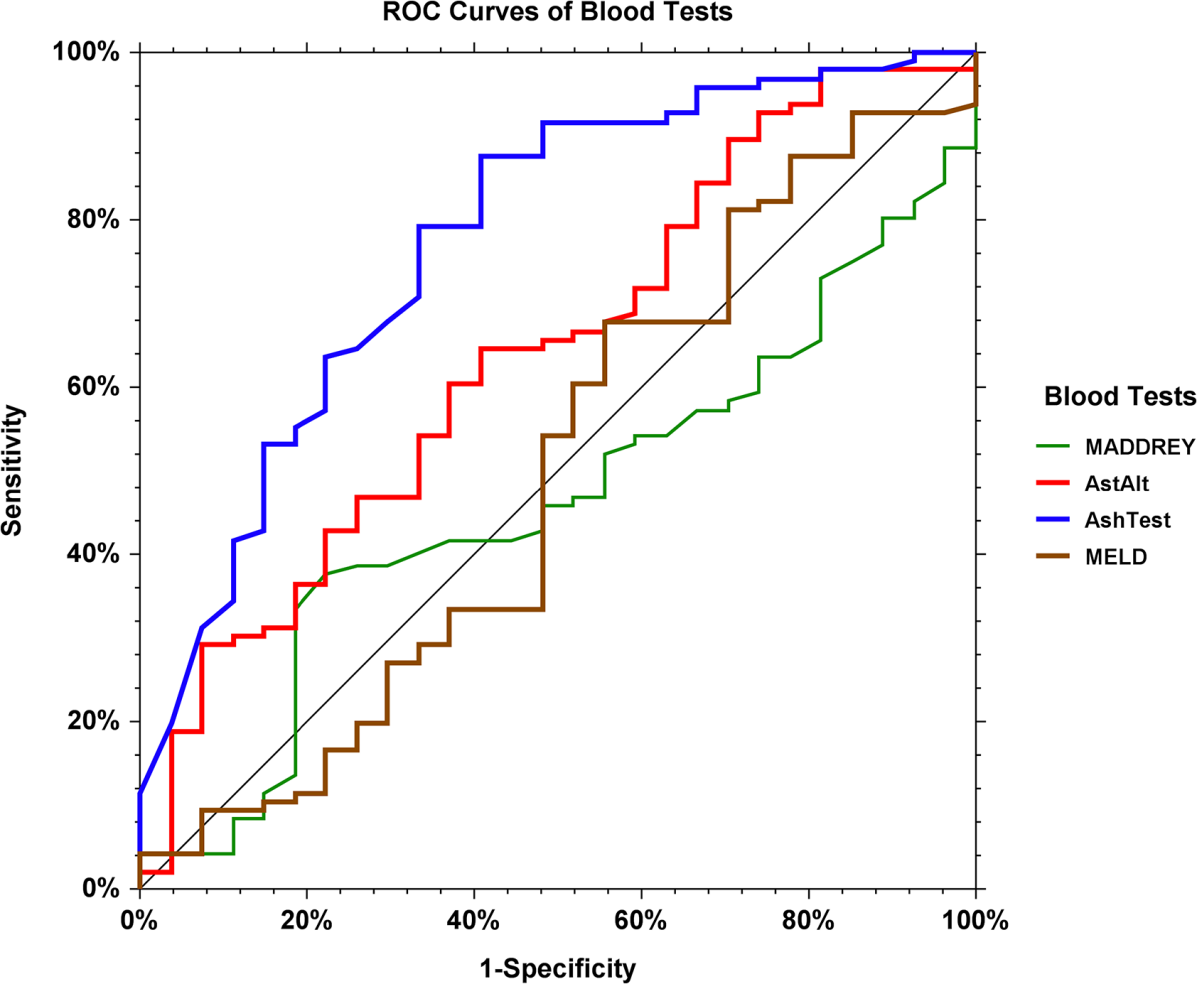
Area under the ROC curves (AUROC) of AshTest versus AST/ALT, Maddrey, and MELD scores. 0.815 (0.639–0.910), 0.565 (0.348–0.725), 0.571 (0.448–0.730) and 0.537 (0.327–0.751) respectively, for the diagnosis of at least one histological features of alcoholic hepatitis. All comparisons were significant (P<0.01) between AshTest and AST/ALT, Maddrey and MELD scores.

Using the EASL binary endpoint, AshTest performance was confirmed in this severe patient population. Using the predetermined cutoff of 0.50 for AshTest, the sensitivity of AshTest was 88.8% and its specificity was 48.0%; the likelihood ratio for AshTest was 1.67 (95%CI 1.20–2.70), and the positive predictive value was 87.9%. The AUROC was 0.803 (95%CI 0.684–0.881), significantly higher than the AST/ALT AUROC [0.603 (0.462–0.714); P<0.001]. AshTest AUROCs varied according to the binary endpoint from 0.777 (0.651–0.861) for presence for 3 lesions, to 0.815 (0.639–0.910) for presence of any single activity lesion.**(Table 2)(Fig 2)**

**Table 2:** Performance of AshTest for the diagnosis (binary) and severity (ordinal) of alcoholic steatohepatitis (n = 123).

Outcome	ASH according to elementary features combination				Ballooning		PMN		Mallory	
Binary or score	Binary EASL Ballooning PMN Steatosis	Binary Sensitive Ballooning or PMN or Mallory	Binary Specific Ballooning PMN Mallory	ASH Score (0–3)	Binary	Score (0–3)	Binary	Score (0–3)	Binary	Score (0–3)
Method	Auroc	Auroc	Auroc	nonBinROC	Auroc	nonBinROC	Auroc	nonBinROC	Auroc	nonBinROC
AshTest m (SE)	0.803 (0.049)	0.815 (0.066)	0.777 (0.053)	0.902 (0.017)	0.773 (0.067)	0.859 (0.020)	0.795 (0.063)	0.878 (0.017)	0.801 (0.053)	0.863 (0.020)
AST/ALT	0.603 (0.064)	0.565 (0.096)	0.641 (0.060)	0.833 (0.023)	0.605 (0.072)	0.808 (0.023)	0.625 (0.078)	0.787 (0.026)	0.634 (0.071)	0.798 (0.028)
P-value AshTest vs AST/ALT	P<0.001	P<0.001	P = 0.03	P = 0.01	P = 0.01	P = 0.09	P = 0.01	P = 0.003	P = 0.02	P = 0.05

AshTest had significant higher AUROCs than AST/ALT for all scores and lesions. NonBinROC is the Obuchowski measure, the non-binary estimate of ordinal test performance. AshTest had significant Obuchowski measures than AST/ALT for ASH scores and for PMN and Mallory.

Using the ordinal endpoint, AshTest performance was also significant versus AST/ALT [Obuchowski measure = 0.902 (0.017) vs. 0.833 (0.023), P = 0.01].(**S3 Table**) The main differences were observed for non-ASH vs. minimal ASH and for moderate vs. severe ASH.(**Table 3**) Using pathologist conclusion as endpoint, AshTest performances were similar than using EASL endpoint.(**S4 Table**) AshTest had also higher performance for histological ASH scores than MaddreyDF and MELD score.(Fig 2)(**S5 Table)**.

**Table 3:** Concordance between ASH grades presumed by AshTest and by biopsy.

AshTest (4 grades)	Histological ASH score (4 grades)				
	No ASH Score = 0	Minimal Score = 1–2	Moderate Score = 3–5	Severe Score = 6–9	Total
No ASH 0-≤0.17	2	2	2	0	6
Minimal >0.17–0.5535	4	5	10	1	20
Moderate >0.5535-≤0.78	1	3	10	5	19
Severe >0.78–1	2	3	36	37	78
Total	9	13	58	43	123

The histological score is the sum of the 3 elementary lesion grades: Ballooning, PMN and Mallory bodies.

### Discordance analyses

Concordance between the histological scores and AshTest scores was significant (P<0.0001) and was rated as moderate to fair (wKappa = 0.48; 95% CI, 0.34–0.63). Discordance was minimal, and a difference of only one grade was found in 60/123 (49%) patients, and of 2 grades or more in only 9/123 (7.3%) patients.(**S6 Table**) Two or three grades discordance was attributed to failure of biopsy (false negative) in five (4%) cases (including the only "three-grade discordances"); the lengths ranged from 10 to 14 mm, and there were 2 to 12 fragments. Discordance was attributable to AshTest (false negative) in three cases, as the biopsy was 20 mm or longer, and the ApoA1 was not decreased, as is usually observed in histological ASH. In the remaining cases, the cause of discordance could not be determined, though cardiac insufficiency, cirrhosis and sinusoid dilatation, as well as minimal PMN and ballooning were suspected. Univariate comparisons between the 54 concordant cases and the 69 discordant cases identified the associated factors as the clinical non-severity of ASH (P<0.0001), the number of fragments (P = 0.02) and the inferior vena cava pressure (P = 0.03). In multivariate analysis the only factor associated with discordance was the clinical non-severity of ASH (P<0.001).(**S7 Table**)

In a "worse scenario" for AshTest, assuming no failure for biopsy, the failure rate due to the test was 49% for one ASH-score grade and 7% for two grades. In a best scenario focusing on 2+ grades discordances and assuming high risk of failure for biopsy, the failure attributable rate of AshTest was 2.4% (3/123), 4.1% (5/123) for biopsy and unknown in the remaining case (0.8%).

Finally, due to the severity of liver disease all these patients were considered for treatment by corticosteroids. Based on AshTest only, or based on biopsy only, 109 (89%) and 88 (72%) patients would have been treated respectively.(S3 File)(**S7 Table)**

### Prognostic performance

In this population that includes only severe end-stage liver diseases, only the Maddrey discriminant function had a significant prognostic value in multivariate analysis [Risk ratio = 1.01 (95%CI 1.006–1.020) P = 0.0005]. AshTest, AST/ALT, MELD, AHHS and hemodynamic measurements had no prognostic value.(**S8 Table**). There was no significant association between AshTest and gradient, VCI and OD pressure.(**S9 Table**).

## Discussion

The results confirmed the diagnostic performance of AshTest in the appropriate context of use, patients with the most severe form of alcoholic steatohepatitis, on a greater number of patients and using both a binary and four-grade scoring system.[3] Therefore the guidelines that recommended transjugular liver biopsy to prove the presence of histological ASH before starting treatment such as corticosteroids need to be challenged.[1,2] As for other tests, the benefit-risks of AshTest versus transjugular biopsy will be discussed according to their respective advantages and limitations.[17,18]

### Limitations of AshTest

The three main limitations of AshTest are the non-independent status of the authors with possible conflicts of interest, the relatively low availability of the test compared with non-patented tests such as the AST/ALT ratio, and the relatively small number of patients included.

We acknowledge that a fully independent validation of AshTest is still missing. Contrary to FibroTest, which has been validated by several independent studies in alcoholic liver disease [19,20,21,22,23] since the first publication in 2006, no studies have re-validated AshTest. The lack of funding in alcoholic liver disease is certainly one explanation.[24] We therefore decided to perform another validation in the specific context of use of suspected severe histological ASH in order to support the treatment decision.

The relative non-availability of AshTest/AshFibroSure is increasingly less of a limitation, as AshTest uses the same components as FibroTest/FibroSure (plus AST), which is now prescribed and available in more than 50 countries. FibroTest also enables confirmation of the fibrosis stage with 100% positive predictive value in the present study for cirrhosis, including in the case of cardiovascular insufficiency.

### Advantages of biopsy

The recognized advantages of transjugular liver biopsy are its status as the gold standard; its direct assessment of elementary lesions; the simultaneous assessment of the HVPG, which was previously validated as a prognostic index in cirrhotic patients; and its potential ability to diagnose associated causes, such as nodular hyperplasia or heart failure.[24,25,26]

The utility of HVPG in our population was unclear, as its prognostic value was not significant and was lower than that of the Maddrey DF (**S8 Table**). Biopsy was useful in only one patient, showing mixed liver lesions including sinusoidal dilatation, cirrhosis, minimal PMN and minimal ballooning. The pathologist diagnosed cardiac-related liver disease without obvious histological ASH. This patient probably had a mixed cause of cirrhosis, heart failure with an elevated gradient (23 mmHg) together with a high (19 mmHg) inferior vena cava pressure (IVC), and minimal histological ASH. Interestingly, we observed that 31 out of 97 patients (32%) had IVC pressures greater than 15 mmHg, suggesting frequent undiagnosed right heart failure in these patients. These possible relationships between clinical or histological ASH and alcoholic cardiovascular disease should be explored.

### Limitations of biopsy

Transjugular biopsy has a 0.18% probability of resulting in mortality, with an additional 1.27% risk of serious adverse event, as observed in our population (0.8%).[26]

In the severe clinical ASH context of use, the other major biopsy limitations were the small specimen length, as well as the large number of fragments. Indeed, the mean number of fragments was significantly higher in discordant versus concordant patients (11 vs. 8). The analyses of severe discordance (2 grades or more) suggested that low quality specimens were associated with 5 false negatives of biopsy and 3 false negatives of AshTest. Therefore, and as observed in NAFLD [27] and chronic hepatitis C,[17,28] liver biopsy is far from being a perfect reference test in patients with severe clinical ASH [8]. This risk of false negatives due to sampling error was also illustrated by one of our patients who was still an active drinker, and who had undergone two biopsies 9 months apart, one without histological ASH (22 mm, 12 fragments) and one with severe histological ASH (26 mm, 4 fragments). Sampling error should be at least equal to those we describe for NASH.[27]

Interobserver variability is another cause of false positives or false negatives. Even in 392 drinkers without severe histological ASH, we observed moderate or fair concordance (binary outcome) between two observers using intercostal biopsy specimens, which were of better quality than those from the transjugular route: only 17% were fragmented and 88% were longer than 10 mm.[8]

In a recent prognostic study of severe histological ASH, similar limitations of biopsy were observed, including a relatively limited number of subjects despite the international multicenter design (121 in the first set, and 96 in the updated set), small specimen length (median 6 mm) and substantial interobserver variability (non-weighted kappa concordance coefficient ranging from 0.46 to 0.65) compared to the usual coefficient of variation of blood tests lower than 10%.[9,17]

### Costs

The cost of transjugular biopsy is estimated at £1,500 (€1900), and requires an overnight stay and possible transportation costs.

The cost of AshTest/Ash-FibroSure varied according to countries and health care systems, from 30 euros to 487 dollars, but 50 Euros for FibroMax (which combines FibroTest, ActiTest, SteatoTest, AshTest and NashTest) is the competitive median price of patented blood liver tests.[29]

### Advantages of AshTest

The four advantages of AshTest compared with biopsy are its absence of adverse events, its rapid assessment, its lower variability and lower cost. All components of AshTest can be measured on a single analyzer, and the algorithms, including the security algorithm directly integrated in the biochemistry routine process, enable the results to be obtained in less than 2 hours in our department.

We acknowledge that there is no recognized reference blood test for ASH, and that AST/ALT ratio is considered to be reflection of alcoholic liver disease in general and not specific for diagnosis of alcoholic hepatitis. However AST/ALT ratio was the only "routine" biochemical test discussed in the diagnostic paragraph of EASL guidelines.[2] One original result of the present study was to demonstrate that the performance AshTest was still higher than AST/ALT ratio in this severe population with Maddrey-DF >32. AshTest versus non-patented tests, such as the AST/ALT ratio, has a higher diagnostic performance for the binary and ordinal severity scores of histological ASH. Its improved performance was confirmed for the overall histological diagnosis, as well as for the elementary lesions, particularly for PMN (**Table 2**). This overall greater performance was mainly due to the ability to discriminate between moderate and minimal histological ASH (**S3 Table**). The advantage of the AST/ALT ratio is its cost and availability. Another advantage of the AshTest versus the AST/ALT ratio is the possibility of simultaneously assessing the stage of fibrosis using FibroTest, and the steatosis grade using SteatoTest in the FibroMax combination using the same sample. A combination of white blood cell and platelet counts had significant performance for the binary diagnosis of histological ASH, in one study.[30] However these results should be interpreted with cautious due to retrospective design, the small sample size with only 58 patients, of which 43 had ASH as confirmed with a liver biopsy; therefore the specificity was assessed in only 5 patients without histological ASH.[30]

### Limitations of the study design

In our first AshTest validation, we recognized that the small number of patients with severe histological ASH (53 out of 225 patients and 299 controls), was a limitation.[3] Therefore this second validation provides, important additional evidence based on the same outcomes and cutoffs, and in the appropriate context of use, a new population of 123 patients who were predetermined as candidates for corticosteroid treatment.

We focused on the diagnosis of histological ASH and not on its prognostic performance, as the AshTest was developed from its inception for diagnosis. We acknowledge that the presence of megamitochondria was not prospectively predetermined with the appropriate recommendations, such as the high magnitude power field (600 vs400).[9] Since all of the patients had cirrhosis with clinically severe acute alcoholic hepatitis, the population may not show sufficient variation to appropriately evaluate prognostic significance.

Patients were consecutive suspected severe ASH, clinically defined, and all of them had cirrhosis, which could be also viewed as a limitation. The first validation validated AshTest in a population with a broad spectrum of clinical ASH. In this second validation the population was patients with cirrhosis and severe ASH, which is the target of an unmet need: non-invasive biomarker as treatment such as corticosteroid are discussed and they needed transjugular biopsy. Patients with cirrhosis represented 82% of patients with suspected ASH in of a recent multicenter study with the higher odds ratio for short-term survival.[9]

### Better validation of biomarkers with better definition of ASH

Finally according to the limitation of biopsy it seems fair to simplify the definition the ASH (necro-inflammatory activity) from the non-activity features of ALD (steatosis, fibrosis), as it had been achieved for elementary histological features of chronic viral hepatitis 21 years ago with the METAVIR scoring system,[4] and recently for NAFLD with the SAF score.[6] In ALD new tests should be developed to predict activity, independently of steatosis and fibrosis, including not only the binary diagnosis but also the ordinal diagnosis of severity stages. We believe that the EASL definition is no more appropriate, as it combined steatosis with activity features (ballooning and PMN).

## Conclusion

These results confirm the performance of AshTest in 123 new patients with suspected severe clinical ASH and within the specific context of corticosteroid treatment use. AshTest has limitations, including a 2–7% risk of two grades misclassification, but is an appropriate non-invasive alternative to transjugular liver biopsy, allowing an estimate of histological activity grades. It could be included in updated algorithms for the diagnosis and treatment of histological and clinical ASH.[1,2,25,29]

## Supporting Information

S1 File Histological methods and details of elementary lesion scores and different ASH definitions and grades. (DOCX)

S2 File Statistical methods, Obuchowski measure. (DOCX)

S3 File Discordances in treatment according to discordance in diagnosis. (DOCX)

S1 Table Histological methods and details of elementary lesion scores and different ASH definitions and grades (DOCX)

S2 Table Comparison of AshTest components between patients with or without sepsis at baseline. (DOCX)

S3 Table Performance (Obuchowski measure NonBinROC) of AshTest for the diagnosis of histological ASH scores versus AST/ALT, Maddrey and MELD scores. Over all and stage by stage performances.
(DOCX)

S4 Table Performance of AshTest for the diagnosis (binary) and severity (ordinal) of alcoholic steatohepatitis (n = 123), according to EASL or a central pathologist conclusion. (DOCX)

S5 Table Performance of AshTest for the diagnosis (binary) and severity (ordinal) of histological alcoholic steatohepatitis (ASH) (n = 123). Details versus Maddrey and MELD scores.
(DOCX)

S6 Table Details of patients with discordance of 2 grades or more between AshTest and biopsy. (DOCX)

S7 Table Characteristics of patients non discordant (one grade or more) and discordant for the diagnosis of severe ASH. (DOCX)

S8 Table Prognostic performance of AshTest. (DOCX)

S9 Table Hemodynamics and AshTest score. (DOCX)

S10 Table AshTest STARD-Checklist for reporting of studies of diagnostic accuracy (DOCX)
