# Supplementary material for: Validation of AshTest as a Non-Invasive Alternative to Transjugular Liver Biopsy in Patients with Suspected Severe Acute Alcoholic Hepatitis
Source: PLoS One. 2015 Aug 7;10(8):e0134302. doi: 10.1371/journal.pone.0134302 (PMC4529115; doi:10.1371/journal.pone.0134302)
Supplement: S1 File — (DOCX) [file pone.0134302.s001.docx]

**S1 File. Histological methods and details of elementary lesion scores and different ASH definitions and grades. [References are those of the manuscript]:**

In histological ASH there are no consensual binary definitions or (semi) quantitative scoring systems.[1,2] In the EASL [European Association for the Study of the Liver] guidelines, histological ASH is defined by "the coexistence of steatosis, hepatocyte ballooning and an inflammatory infiltrate with polymorphonuclear neutrophils (PMN). The presence of Mallory-Denk's bodies, and mega-mitochondria, although not specific to ASH, are often associated with these elementary lesions."[2]

Scoring procedures that focus on the main "independent" elementary lesions, as proposed for chronic viral hepatitis [4] or non-alcoholic steatohepatitis (NASH) [5,6], could be readily adapted for use in histological ASH.[7] We previously used such scoring systems in these patients with ASH by accumulating the grades of the elementary ASH lesions.[3,8]

For consistency with these recommendations and with the standard definition of EASL (combining steatosis and activity), we used two primary endpoints: one binary (the presence or absence of ASH as defined by EASL) and one non-binary (ordinal according to an activity score) endpoint.

In sensitivity analyses, the following four other binary definitions of ASH were used: the pathologist’s main conclusion, the presence of two elementary lesions, the presence of ballooning and PMN, the presence of at least one elementary lesion, such as ballooning, PMN or Mallory bodies and the presence of all three lesions.

The non-binary primary endpoint, as used in recent scoring systems validated in viral hepatitis or NAFLD, was the sum of the three elementary lesion grades (none, minimal, moderate, severe; from 0 to 3), resulting in a 4-grade severity score: H0, no ASH; H1, minimal ASH (score 1-2); H2, moderate ASH (score 3-5); and H3, severe ASH (score 6-9). In sensitivity analyses, the severity of histological ASH, also given by the pathologist in four grades in his conclusion, was also used to evaluate the test performance.

At the end of our prospective evaluation of elementary lesions by the centralized pathologist, an international multicenter study proposed, as a prognosis index, a new histological classification of ASH (the AHHS [Alcoholic Hepatitis Histologic score], from 0 to 9: mild 0-3; intermediate 4-5; severe 6-9). It also included stage of fibrosis (no fibrosis or portal fibrosis 0; expansive fibrosis 0; bridging fibrosis or cirrhosis +3), bilirubinostasis (no or hepatocellular only 0; canalicular or ductular +1; canalicular or ductular plus hepatocellular +2); PMN infiltration (no/mild +2; severe 0) and megamitochondria (no +2, yes 0). As these lesions were also part of our histological report, the diagnostic and prognostic performances of this score were also "retrospectively" compared to AshTest.[9]

Steatosis was scored from 0 to 100 according to the percentage of hepatocytes with macro- or microsteatosis. Fibrosis was staged with a scoring system adapted from the METAVIR score using a scale from F0 to F4: F0, no ﬁbrosis; F1, ﬁbrosis without septa; F2, few septa; F3, numerous septa without cirrhosis; and F4, cirrhosis. [10]
