# Supplementary material for: Validation of AshTest as a Non-Invasive Alternative to Transjugular Liver Biopsy in Patients with Suspected Severe Acute Alcoholic Hepatitis
Source: PLoS One. 2015 Aug 7;10(8):e0134302. doi: 10.1371/journal.pone.0134302 (PMC4529115; doi:10.1371/journal.pone.0134302)
Supplement: S2 File — (DOCX) [file pone.0134302.s002.docx]

**S2 File. Statistical methods, Obuchowski measure**

Obuchowski measure is a multinomial version of the AUROC. With N (=4) categories of the gold standard outcome (histological ASH grade) and AUROCst (the estimate of the AUROC of diagnostic tests for differentiating between categories s and t) the Obuchowski measure is a weighted average of the N(N–1)/2 (=6) different AUROCst corresponding to all the pairwise comparisons between two of the N categories. Each pairwise comparison has been weighted to take into account the distance between activity grades (i.e. the number of units on the ordinal scale). A penalty function proportional to the difference in scoring system units between grads was defined, being 0.33 when the difference between grades was 1, 0.67 when the difference was 2, and 1 when the difference was 3. The Obuchowski measure can be interpreted as the probability that the test will correctly rank two randomly chosen patient samples from different ASH grades according to the weighting scheme, with a penalty for misclassifying patients. The overall Obuchowski measure is not equivalent to a usual AUROC curve, as the measurements are weighted according to the distance between grades. Sensitivity analyses compared AUROCs according to biopsy specimen length and the number of fragments, and according to several definitions of histological ASH based on combinations of elementary lesions.
