# Supplementary material for: Validation of AshTest as a Non-Invasive Alternative to Transjugular Liver Biopsy in Patients with Suspected Severe Acute Alcoholic Hepatitis
Source: PLoS One. 2015 Aug 7;10(8):e0134302. doi: 10.1371/journal.pone.0134302 (PMC4529115; doi:10.1371/journal.pone.0134302)
Supplement: S3 File — (DOCX) [file pone.0134302.s003.docx]

**S3 File. Discordances in treatment according to discordance in diagnosis:**

Finally due to the severity of liver disease all these patients were considered for treatment by corticosteroids. Based on AshTest only, or based on biopsy only, 109 (89%) and 88 (72%) patients would have been treated respectively

A total of 117 patients have been treated by corticosteroid. Among them 80 were concordantly diagnosed by AshTest and biopsy. Among the 37 discordantly diagnosed, 8 were finally considered as false positive of biopsy and 29 false negative of biopsy.

Based on AshTest only or biopsy only, no change would have occurred among the 6 not treated by corticosteroid. Four patients were concordantly diagnosed, one without ASH and the remaining three with ASH not treated due to one rapid death after admission, and two sepsis including one death. The remaining two discordantly diagnosed were considered as false negative of biopsy but not treated; one was not treated, as he was non-responder to 3 previous treatments by corticosteroids and the other one died rapidly after admission.
