# Supplementary material for: Validation of AshTest as a Non-Invasive Alternative to Transjugular Liver Biopsy in Patients with Suspected Severe Acute Alcoholic Hepatitis
Source: PLoS One. 2015 Aug 7;10(8):e0134302. doi: 10.1371/journal.pone.0134302 (PMC4529115; doi:10.1371/journal.pone.0134302)
Supplement: S1 Table — (DOCX) [file pone.0134302.s004.docx]

**S1 Table. Histological methods and details of elementary lesion scores and different ASH definitions and grades**

| Elementary lesions, scores and definitions | n/123 (percent) |
| --- | --- |
| *Ballooning* |  |
| None | 13 (10.6%) |
| Minimal | 40 (32.5%) |
| Moderate | 37 (30.1%) |
| Severe | 33 (26.8%) |
| *PMN* |  |
| None | 18 (14.6%) |
| Minimal | 59 (48.0%) |
| Moderate | 23 (18.7%) |
| Severe | 23 (18.7%) |
| *Mallory bodies* |  |
| None | 21 (17.1%) |
| Minimal | 57 (46.3%) |
| Moderate | 10 (8.1%) |
| Severe | 35 (28.5%) |
| *Cumulative severity score* |  |
| None (0) | 9 (7.3%) |
| Minimal (1-2) | 13 (10.6%) |
| Moderate (3-5) | 58 (47.2%) |
| Severe (6-9) | 43 (35.0%) |
| *Pathologist severity score* |  |
| None | 17 (13.8%) |
| Minimal | 35 (28.5%) |
| Moderate | 33 (26.8%) |
| Severe | 38 (30.9%) |
| *Histological ASH definition* |  |
| Ballooning and PMN and Mallory | 96 (78.0 %) |
| Ballooning and PMN and steatosis (EASL) | 98 (79.7%) |
| Mallory bodies | 103 (83.7%) |
| Ballooning and PMN | 101 (82.1%) |
| Ballooning | 110 (89.4%) |
| PMN | 105 (85.4%) |
| Pathologist binary conclusion | 106 (86.2%) |
| Ballooning or PMN or Mallory | 112 (91.1%) |
