# Supplementary material for: Validation of AshTest as a Non-Invasive Alternative to Transjugular Liver Biopsy in Patients with Suspected Severe Acute Alcoholic Hepatitis
Source: PLoS One. 2015 Aug 7;10(8):e0134302. doi: 10.1371/journal.pone.0134302 (PMC4529115; doi:10.1371/journal.pone.0134302)
Supplement: S2 Table — (DOCX) [file pone.0134302.s005.docx]

**S2 Table: Comparison of AshTest components between patients with or without sepsis at baseline.**

| Baseline value m (SD) | No-Sepsis (n=100) | Sepsis (n=23) | P-value (Mann-Whitney) |
| --- | --- | --- | --- |
| AshTest (0.00-1.00) | 0.73 (0.28) | 0.89 (0.12) | 0.02 |
| Haptoglobin g/L | 0.32 (0.37) | 0.48 (0.51) | 0.28 |
| Alpha2 Macroglobulin g/L | 1.99 (0.70) | 1.84 (0.74) | 0.29 |
| Apolipoprotein A1 g/L | 0.45 (0.34) | 0.24 (0.15) | 0.003 |
| Total bilirubin mmol/L | 172 (136) | 202 (127) | 0.11 |
| GGT IU/L | 265 (436) | 270 (321) | 0.84 |
| FibroTest (0.00-1.00) | 0.96 (0.04) | 0.97 (0.03) | 0.10 |
| Age mean (sd) | 55.6 (8.8) | 54.4 (9.4) | 0.46 |
| Maddrey function | 62 (30) | 68 (41) | 0.66 |
| MELD score | 20 (6) | 24 (9) | 0.02 |
| ALT | 70 (113) | 57 (36) | 0.81 |
| AST | 156 (158) | 166 (99) | 0.28 |
| AST/ALT | 2.79 (1.51) | 3.09 (1.29) | 0.33 |

The proteins possibly associated with acute or chronic inflammation were not associated with sepsis at baseline, such as haptoglobin and alpha2 -macroglobulin.

Only the MELD prognostic index was associated with sepsis, as well as apoA1 as previously observed.[3]
