# Supplementary material for: Validation of AshTest as a Non-Invasive Alternative to Transjugular Liver Biopsy in Patients with Suspected Severe Acute Alcoholic Hepatitis
Source: PLoS One. 2015 Aug 7;10(8):e0134302. doi: 10.1371/journal.pone.0134302 (PMC4529115; doi:10.1371/journal.pone.0134302)
Supplement: S3 Table — Over all and stage by stage performances. (DOCX) [file pone.0134302.s006.docx]

**S3 Table. Performance (Obuchowski measure NonBinROC) of AshTest for the diagnosis of histological ASH scores versus AST/ALT, Maddrey and MELD scores. Over all and stage by stage performances.**

|  | **Alcoholic Hepatitis scores** | |  | |  | |  | |
| --- | --- | --- | --- | --- | --- | --- | --- | --- |
| **Stages*** | **Cumulative elementary scores (4 classes)** | | | | **Pathologist score (4 classes)** | | | |
|  | AshTest | AST/ALT | Maddrey DF | MELD | AshTest | AST/ALT | Maddrey DF | MELD |
| All | 0.902 (0.017) | 0.833 (0.023) | 0.748 (0.023) | 0.786 (0.024) | 0.854 (0.020) | 0.793 (0.025) | 0.716 (0.024) | 0.747 (0.027) |
| H0 vs. H1 | 0.564 (0.134) | 0.547 (0.138) | 0.625 (0.120) | 0.628 (0.132) | 0.709 (0.077) | 0.640 (0.083) | 0.568 (0.084) | 0.521 (0.098) |
| H0 vs. H2 | 0.794 (0.090) | 0.592 (0.113) | 0.595 (0.117) | 0.626 (0.128) | 0.766 (0.076) | 0.571 (0.093) | 0.565 (0.084) | 0.520 (0.095) |
| H0 vs. H3 | 0.907 (0.063) | 0.694 (0.108) | 0.530 (0.114) | 0.595 (0.122) | 0.868 (0.055) | 0.711 (0.076) | 0.543 (0.081) | 0.500 (0.088) |
| H1 vs. H2 | 0.752 (0.076) | 0.546 (0.088) | 0.533 (0.063) | 0.541 (0.063) | 0.548 (0.071) | 0.593 (0.070) | 0.507 (0.072) | 0.558 (0.072) |
| H1 vs. H3 | 0.894 (0.054) | 0.687(0.069) | 0.619 (0.073) | 0.502 (0.076) | 0.701 (0.061) | 0.587 (0.067) | 0.601 (0.067) | 0.526 (0.069) |
| H2 vs. H3 | 0.700 (0.052) | 0.634 (0.056) | 0.588 (0.071) | 0.527 (0.073) | 0.667 (0.066) | 0.675 (0.065) | 0.602 (0.069) | 0.500 (0.070) |

*The histological score is the sum of the 3 elementary lesion grades: Ballooning, PMN and Mallory bodies

H0: no lesion, H1: minimal, H2 moderate, H3 severe
