# Supplementary material for: Validation of AshTest as a Non-Invasive Alternative to Transjugular Liver Biopsy in Patients with Suspected Severe Acute Alcoholic Hepatitis
Source: PLoS One. 2015 Aug 7;10(8):e0134302. doi: 10.1371/journal.pone.0134302 (PMC4529115; doi:10.1371/journal.pone.0134302)
Supplement: S4 Table — (DOCX) [file pone.0134302.s007.docx]

**S4 Table. Performance of AshTest for the diagnosis (binary) and severity (ordinal) of alcoholic steatohepatitis (n=123), according to EASL or a central pathologist conclusion.**

| **Outcome** | **ASH according to elementary features** | | **ASH according to pathologist conclusion** | | |
| --- | --- | --- | --- | --- | --- |
| Binary or score | EASL Binary | Score (0-3) | Binary | Score (0-3) | |
| Method | Auroc | nonBinROC | Auroc | nonBinROC | |
| AshTest m (SE) | 0.803 (0.049) | 0.902 (0.017) | 0.807 (0.059) | 0.854 (0.020) | |
| AST/ALT | 0.603 (0.096) | 0.833 (0.023) | 0.653 (0.074) | 0.793 (0.025) | |
| Significance AshTest vs AST/ALT | P<0.001 | P=0.01 | P=0.02 | | P=0.05 |

AshTest had significant higher AUROCs than AST/ALT for all scores and lesions.

NonBinROC is the Obuchowski measure, the non-binary estimate of ordinal test performance. AshTest had significant Obuchowski measures than AST/ALT for ASH scores and for PMN and Mallory.
