# Supplementary material for: Validation of AshTest as a Non-Invasive Alternative to Transjugular Liver Biopsy in Patients with Suspected Severe Acute Alcoholic Hepatitis
Source: PLoS One. 2015 Aug 7;10(8):e0134302. doi: 10.1371/journal.pone.0134302 (PMC4529115; doi:10.1371/journal.pone.0134302)
Supplement: S5 Table — Details versus Maddrey and MELD scores. (DOCX) [file pone.0134302.s008.docx]

**S5 Table. Performance of AshTest for the diagnosis (binary) and severity (ordinal) of histological alcoholic steatohepatitis (ASH) (n=123). Details versus Maddrey and MELD scores.**

| **Outcome** | **ASH according to elementary features** | | **ASH according to pathologist conclusion** | | **Ballooning** | | **PMN** | | **Mallory** | |
| --- | --- | --- | --- | --- | --- | --- | --- | --- | --- | --- |
| **Binary or score** | **Binary EASL** | **Score (0-3)** | **Binary** | **Score (0-3)** | **Binary** | **Score (0-3)** | **Binary** | **Score (0-3)** | **Binary** | **Score (0-3)** |
| Method | Auroc | nonBinROC | Auroc | nonBinROC | Auroc | nonBinROC | Auroc | nonBinROC | Auroc | nonBinROC |
| AshTest m(SE) | 0.803 (0.049) | 0.902 (0.017) | 0.807 (0.059) | 0.854 (0.020) | 0.773 (0.067) | 0.859 (0.020) | 0.795 (0.063) | 0.878 (0.017) | 0.801 (0.053) | 0.863 (0.020) |
| Maddrey | 0.565 (0.097) | 0.748 (0.023) | 0.501 (0.073) | 0.716 (0.024) | 0.559 (0.067) | 0.718 (0.023) | 0.475 (0.078) | 0.725 (0.026) | 0.474 (0.067) | 0.717 (0.024) |
| MELD | 0.576 (0.108) | 0.786 (0.024) | 0.508 (0.084) | 0.747 (0.027) | 0.608 (0.080) | 0.787 (0.025) | 0.489 (0.083) | 0.764 (0.027) | 0.486 (0.080) | 0.697 (0.031) |
| P AshTest vs Maddrey | <0.01 | <0.01 | <0.01 | <0.01 | 0.04 | <0.01 | <0.01 | <0.01 | <0.01 | <0.01 |
| P AshTest vs MELD | <0.01 | <0.01 | <0.01 | <0.01 | <0.01 | <0.01 | <0.01 | <0.01 | <0.01 | <0.01 |
