# Supplementary material for: Validation of AshTest as a Non-Invasive Alternative to Transjugular Liver Biopsy in Patients with Suspected Severe Acute Alcoholic Hepatitis
Source: PLoS One. 2015 Aug 7;10(8):e0134302. doi: 10.1371/journal.pone.0134302 (PMC4529115; doi:10.1371/journal.pone.0134302)
Supplement: S6 Table — (DOCX) [file pone.0134302.s009.docx]

**S6 Table. Details of patients with discordance of 2 grades or more between AshTest and biopsy.**

| **Case** | **Biopsy** |  |  |  |  | **AshTest** | | **Attribution** |
| --- | --- | --- | --- | --- | --- | --- | --- | --- |
|  | Grade from ASH score (0-9) | Binary Pathologist | Length (mm) | Fragments | Biopsy comment | Value | AshTest comment |  |
|  |  |  |  |  |  |  |  | **3 grades discordance** |
| 1 | 0 No ASH | No ASH | 10 | 5 | Small specimen | 0.96 |  | False negative biopsy |
| 2 | 0 No ASH | No ASH | 15 | 12 | Small specimen Minimal necrosis | 0.83 |  | False negative biopsy |
|  |  |  |  |  |  |  |  | **2 grades discordance** |
| 3 | 1 Minimal | No ASH | 10 | 7 | Small specimen | 0.97 |  | False negative biopsy |
| 4 | 2 Minimal | No ASH | 15 | 2 | Small specimen minimal PMN, and Mallory | 0.91 |  | False negative biopsy |
| 5 | 2 Minimal | No ASH | 25 | 1 | Sinusoid dilatation minimal PMN and ballooning | 0.81 |  | Unknown |
| 6 | 0 No ASH | No ASH | 14 | 2 | Small specimen | 0.58 |  | False negative biopsy |
| 7 | 6 Severe | ASH | 30 | 10 |  | 0.21 | ApoA1 1.08g/L | False negative AshTest |
| 8 | 3 Moderate | ASH | 20 | 15 |  | 0.13 | ApoA1 1.30g/L | False negative AshTest |
| 9 | 4 Moderate | ASH | 25 | 1 |  | 0.09 | ApoA1 0.99g/L | False negative AshTest |
