# Supplementary material for: Validation of AshTest as a Non-Invasive Alternative to Transjugular Liver Biopsy in Patients with Suspected Severe Acute Alcoholic Hepatitis
Source: PLoS One. 2015 Aug 7;10(8):e0134302. doi: 10.1371/journal.pone.0134302 (PMC4529115; doi:10.1371/journal.pone.0134302)
Supplement: S7 Table — (DOCX) [file pone.0134302.s010.docx]

**S7 Table. Characteristics of patients non discordant (one grade or more) and discordant for the diagnosis of severe ASH.**

| Baseline characteristics | Non-discordant | n | Discordant | n | P-value |
| --- | --- | --- | --- | --- | --- |
| AshTest | 0.82 (0.23) | 54 | 0.71 (0.28) | 69 | 0.02 |
| AST/ALT | 3.1 (1.7) | 54 | 2.6 (1.2) | 69 | 0.07 |
| FibroTest | 0.96 (0.04) | 54 | 0.96 (0.04) | 69 | 0.54 |
| Maddrey | 61 (30) | 54 | 64 (33) | 69 | 0.51 |
| MELD | 21 (8) | 54 | 21 (6) | 69 | 0.77 |
| Hemodynamics |  |  |  |  |  |
| Portal gradient (mm Hg) | 18.2 (8.1) | 40 | 16.1 (6.4) | 57 | 0.62 |
| Inferior vena cava (mm Hg) | 15.4 (7.8) | 40 | 12.4 (5.1) | 57 | 0.03 |
| Right auricular (mmHg) | 8.7 (6.4) | 40 | 7.9 (5.0) | 57 | 0.97 |
| Biopsy |  |  |  |  |  |
| Number of fragments | 8.8 (4.6) | 54 | 11.4 | 69 | 0.03 |
| Length specimen (mm) | 14.6 (5.5) | 54 | 15.9 (6.5) | 69 | 0.30 |
| ASH binary outcome | 94 (90.4%) | 54 | 13 (68.4%) | 69 | 0.02 |
| Ash score (0-9) | 6.2 (2.7) | 54 | 3.4 (1.7) | 69 | <0.0001 |
| Steatosis (%) | 46 (26) | 54 | 38 (30) | 69 | 0.06 |
