# Supplementary material for: Validation of AshTest as a Non-Invasive Alternative to Transjugular Liver Biopsy in Patients with Suspected Severe Acute Alcoholic Hepatitis
Source: PLoS One. 2015 Aug 7;10(8):e0134302. doi: 10.1371/journal.pone.0134302 (PMC4529115; doi:10.1371/journal.pone.0134302)
Supplement: S8 Table — (DOCX) [file pone.0134302.s011.docx]

**S8 Table. Prognostic performance of AshTest.**

| **Prognostic marker** | **2-year survival** | **Cox (adjusted age, gender)** |
| --- | --- | --- |
|  | % (95% CI) Significance | Risk Ratio (95% CI) Significance |
| **Score ASH biopsy** | P=0.17 | 1.02 (0.94-1.11) P=0.69 |
| AshScore <= 5 n=80 | 31% (19-43) |  |
| AshScore > 5 n=43 | 55% (38-72) |  |
| **Score AHHS biopsy** | P=0.89 | 1.01 (0.84-1.21) P=0.90 |
| AHHS moderate n=35 | 42% (23-61) |  |
| AHHS high n=88 | 38% (26-50) |  |
| **AshTest** | P=0.62 | 0.71 (0.32-1.59) P=0.40 |
| ≤0.50, n=26 | 31% (12-51) |  |
| >0.50, n=97 | 42% (31-53) |  |
| **AST/ALT** | P=0.99 | 1.04 (0.88-1.22) P=0.64 |
| ≤2, n=36 | 37% (20-55) |  |
| >2, n=87 | 40% (28-52) |  |
| **MELD** | P=0.43 | 1.00 (0.97-1.03) P=0.83 |
| ≤ 20, n=69 | 43% (29-57) |  |
| >20, n=54 | 35% (21-48) |  |
| **Maddrey** | P=0.18 | 1.01 (1.006-1.020) P=0.0005 |
| ≤ 60, n=87 | 43% (31-56) |  |
| > 60, n=36 | 30% (14-46) |  |
| **FibroTest** | P=0.12 | 149 (0.23-1000) P=0.11 |
| 0.80-0.95, n=39 | 42% (25-60) |  |
| 0.95-1.00, n=83 | 36% (24-48) |  |
| **Gradient** | P=0.93 | 1.00 (0.97-1.04) P=0.76 |
| <12 mmHg, n=27 | 35% (13-58) |  |
| ≥ 12 mmHg, n=70 | 41% (28-54) |  |

Cox was adjusted for age and gender if not already included in tests (AshTest, FibroTest)
