# Supplementary material for: Validation of AshTest as a Non-Invasive Alternative to Transjugular Liver Biopsy in Patients with Suspected Severe Acute Alcoholic Hepatitis
Source: PLoS One. 2015 Aug 7;10(8):e0134302. doi: 10.1371/journal.pone.0134302 (PMC4529115; doi:10.1371/journal.pone.0134302)
Supplement: S9 Table — (DOCX) [file pone.0134302.s012.docx]

**S9 Table. Hemodynamics and AshTest score.**

| **AshScore** | **0** | **1** | **2** | **3** | **Variance analysis** |
| --- | --- | --- | --- | --- | --- |
| n | 8 | 11 | 44 | 34 | P value |
| Right auricula | 8.8 (4.0-13.5) | 8.8 (6.3-11.3) | 7.9 (6.0-9.7) | 8.4 (6.4-10.3) | 0.70 |
| Inferior Vena Cava | 12.6 (8.9-12.6) | 13.0 (10.4-15.6) | 12.7 (10.9-14.6) | 15.4 (12.6-18.1) | 0.31 |
| Free Supra Hepatic Vein | 13.1 (9.0-17.3) | 13.9 (10.2-17.6) | 14.6 (12.7-16.4) | 15.2 (12.8-17.6) | 0.83 |
| Wedged Supra Hepatic Vein | 31.5 (24.5-38.5) | 28.2 (22.4-34.0) | 31.0 (28.9-33.0) | 33.4 (28.4-38.4) | 0.50 |
| Gradient | 18.3 (10.6-26.2) | 14.3 (9.6-19.0) | 16.4 (14.2-18.6) | 18.2 (14.6-21.9) | 0.53 |

There were no significant differences between hemodynamics parameters according to AshTest scores.
